# Prominent Remedies in the Recent Epidemic of Influenza*Read before the Homœopathic Medical Society of the County of New York, February 14th, 1895.

**Published:** 1895-04

**Authors:** Edmund Carleton

**Affiliations:** New York City


### PROMINENT REMEDIES IN THE RECENT EPIDEMIC OF INFLUENZA.*

EDMUND CARLETON, M. D., NEW YORK CITY.

This is a brief and hasty exhibit, by request of your chairman, of the remedies most often found indicated, in my practice, in the epidemic of influenza which has recently been so prevalent.

First comes *Rhus-toxicodendron*, with languor; weakness; ill-defined headache; lame, sore eyes; smell and taste abolished; little appetite or thirst; spasmodic, shattering cough, caused by tickling, referred to the larynx and trachea, worse in the evening, aggravated by lying down and by cold drinks; scanty, mucous expectoration; great soreness around the waist from coughing, the patient supporting his sides with his hands and dreading to cough, not only on account of the soreness, but because one paroxysm would incite another; the suppression being sometimes followed in a few minutes by an easy hawking and slight expectoration of slimy mucus; soreness of muscles of body and limbs; chilliness all over, followed by heat, and these often followed by general sweat.

---

*Read before the Homœopathic Medical Society of the County of New York, February 14th, 1895.

One case cured by *Rhus* deserves separate notice. A young man was seized with violent headache, beginning at the occiput and soon becoming general. Prostration was noticed immediately. I saw him about an hour later. He was pale; almost constantly in motion; reclining upon a sofa with his head upon a high pillow, as he could not bear to lie down; feet oftener upon the floor than elsewhere; sore all over; hands cold; nose stuffed; voice weak and altered; oppressed breathing—almost sighing; pulse 58, weak, irregular, and uncertain. Of course, I was thoroughly alarmed; but finding the temperature *per oram* yet normal, concluded to be calm and make a careful selection of the remedy. Thirty minutes after the first teaspoonful, the patient said he felt better. Convalescence followed rapidly. Forty-eight hours from the start he was well.

The sister remedy of *Rhus*—*Bryonia*—was occasionally indicated, when the patient had stitching pains; great thirst for cold water; desire to keep still, and was worse from motion, especially rising.

Next in frequency to *Rhus* was *Pulsatilla* indicated; and it sometimes required close discrimination between them to get the right remedy. Sensorium not so clouded; less headache; more chilliness; not so restless; cough very similar; expectoration thicker and more lumpy; sweat on head and face. In no case did I find the great trinity—chilliness *and* thirstlessness *and* oppression of the chest. One of these would be lacking—at least not acknowledged. It was oftener oppression of chest that was lacking than either of the others; but the minds of some subjects were apparently too anxious to discern this from the remaining chest sensations.

A number of cases had the following combinations: Sneezing; profuse, acrid coryza; profuse, bland lachrymation; spasmodic, barking cough, excited by tickling in the larynx. Better in the cool air, worse in the warm room. *Allium-cepa* cured these cases quickly.

One patient annoyed me considerably before I secured a good picture of his malady. Remembering the rule of Bœnninghausen, to review every case absolutely *de novo* where the prescription had failed, I made a painstaking search, and was rewarded by finding the following symptoms: Nose, between the eyes and extending backward into the head (lachrymal bones), lame, and sore to pressure; white mucous discharge from nose; a similar discharge hawked from the posterior nares, tasting sweetish; tickling in trachea and bronchia; cough in paroxysms, infrequent but long, hard, and exhausting, with expectoration of scanty, white mucus, tasting sweet. Of course, there was but one remedy, *Kali-bichromicum;* and that brought relief in a few hours, complete restoration to health in three days. A single dose on the tongue. Two similar cases appeared later, and were cured with the same remedy.

Other remedies were occasionally indicated; but those already mentioned were required in a large majority of all the cases. The proper selection was always followed by recovery in a few days, provided the patient was obedient to instructions to remain quiet. A few tried to be smart, and consequently had retarded convalescence. I cannot refrain from giving a short sketch of one odd case, and with that will close.

One evening a young lady came hurriedly into my office, weeping, with a request to call at once upon her grandmother, who, the family thought, would soon die. It transpired that she had been sick with "the grip" two weeks, relying upon domestic treatment and the medicines recommended by friends. Notwithstanding all this skill and attention, she grew worse, and all had become thoroughly frightened. I found her propped up in bed, surrounded by the family. It was a case of senile bronchitis, and every breath a rattle. Frequent, hollow cough, without expectoration. Her constant complaint was of nausea, and of little else. *Ipecacuanha* was clearly the remedy, and that was given at once. Despite the most gloomy outlook, she rallied in a few hours, and got well promptly and without interruption. No other remedy was given.

### Discussion.

Dr. Young—Years ago, in Baltimore, I made a careful study of the grip, and found all the symptoms under *Gelsemium.*

That remedy cured all the cases occurring in my practice, which was not extensive. It is the only remedy I have given since, as I find it covers all the cases in all the grip epidemics, and cures them quickly. *Gelsemium* has all the symptoms that have just been read. It is the remedy for the grip.

Dr. Carleton—Different epidemics commonly require different management. It would be unnecessary to state, in an assemblage like this, that homœopaths prescribe, not for the name of the disease, but for the needs of each case. In an epidemic, many individuals require the same remedy. *Sulphur* has more symptoms than any other remedy; but the discriminating physician does not give *Sulphur* for every diseased condition he meets. The virtues of *Gelsemium* are well known, and it has cured some cases observed by me in this epidemic—very likely not so many as at the hands of others. To prove that it is not the universal remedy, it is only necessary to state that in two cases that fell to my lot, I was over-persuaded to give *Gelsemium* by professional brethren, against my own best judgment. Success did not follow, but rather failure, and after a fair trial of *Gelsemium*, *Pulsatilla* cured speedily. Only yesterday, one of our busy prescribers told me that he had given *Pulsatilla* to all his cases of influenza. All were not well-cured, though all finally recovered. *Cinchona* and *Eupatorium-perfoliatum* have their advocates, and I have seen a very few cures by each. But it is manifestly wrong to claim infallibility for any one drug, while individual cases exhibit characteristic differences.

